# Supplementary material for: Controlling the Redox Catalytic Activity of a Cyclic Selenide Fused to 18-Crown-6 by the Conformational Transition Induced by Coordination to an Alkali Metal Ion
Source: Molecules. 2023 Apr 20;28(8):3607. doi: 10.3390/molecules28083607 (PMC10146268; doi:10.3390/molecules28083607)
Supplement: Supplementary file 1 [file molecules-28-03607-s001.zip › molecules-2342396-supplementary.pdf]

Supporting Information

Controlling the redox catalytic activity of a cyclic selenide fused to 18-crown-6 by the conformational transition induced by coordination to an alkali metal

Michio Iwaoka[1,2,*], Hajime Oba[1] and Takeru Ito[1]

[1] Department of Chemistry, School of Science, Tokai University, Hiratsuka-shi, Kanagawa 259-1292, Japan

[2] Institute of Advanced Biosciences, Tokai University, Hiratsuka-shi, Kanagawa 259-1292, Japan

* Correspondence: miwaoka@tokai.ac.jp; Tel.: +81-463-63-4356

Contents

1. ^{1}H, ^{13}C and ^{77}Se NMR and MALDI-TOF-MS spectra for DHS-crowns (1-4).

2. X-ray crystal data for DHS-crown-4 (1), DHS-crown-4·0.5KI·H_2O (5), DHS-crown-4·0.5NaBr·2H_2O (6), DHS-crown-5·0.5KI (7) and DHS-crown-6·KI (8).

3. Changes of NMR spectra for DHS-crown-6 (3) in CH_3OD by complexation with KI.

4. Series of ^{1}H NMR spectra during ^{1}H NMR titration experiments.

5. Series of ^{1}H NMR spectra during the redox catalytic activity assay for DHS-crown-6 (3) and the KCl complex.

6. ^{1}H and ^{77}Se NMR spectra for the selenoxide of DHS-crown-6 (3^{ox}) and the KCl complex.

7. Results of DFT calculation.

1. ^{1}H, ^{13}C and ^{77}Se NMR and MALDI-TOF-MS spectra for DHS-crowns (1-4).

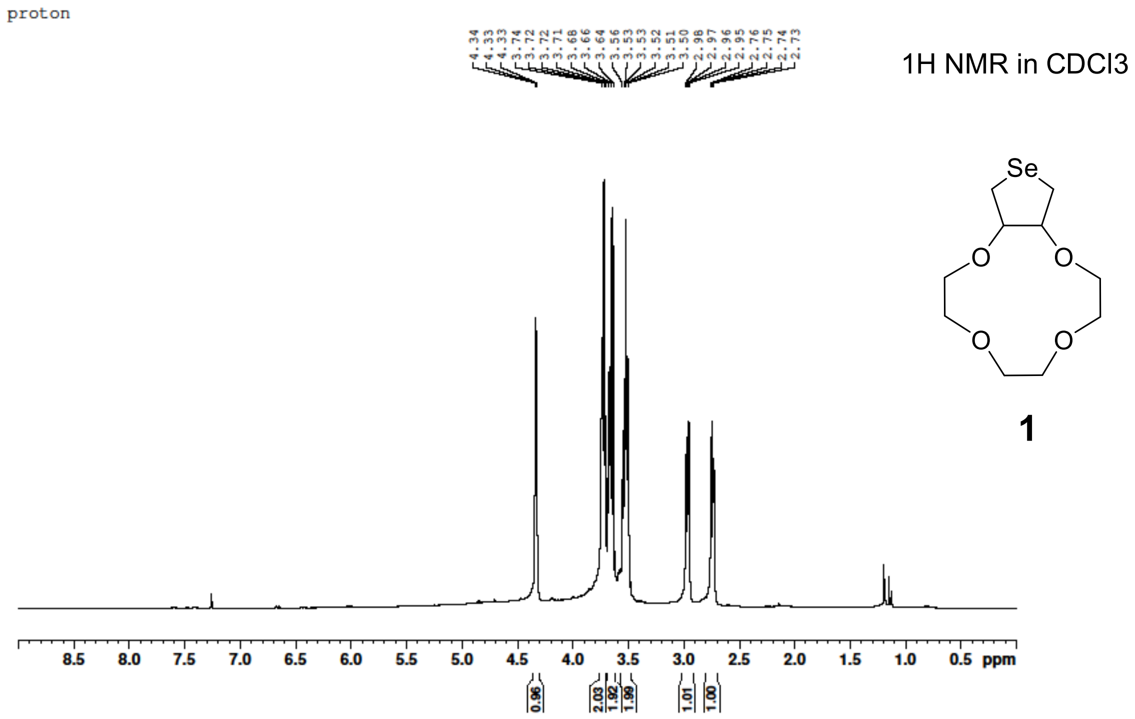

Figure S1. ^{1}H NMR spectrum for DHS-crown-4 (**1**) in $CDCl_3$.

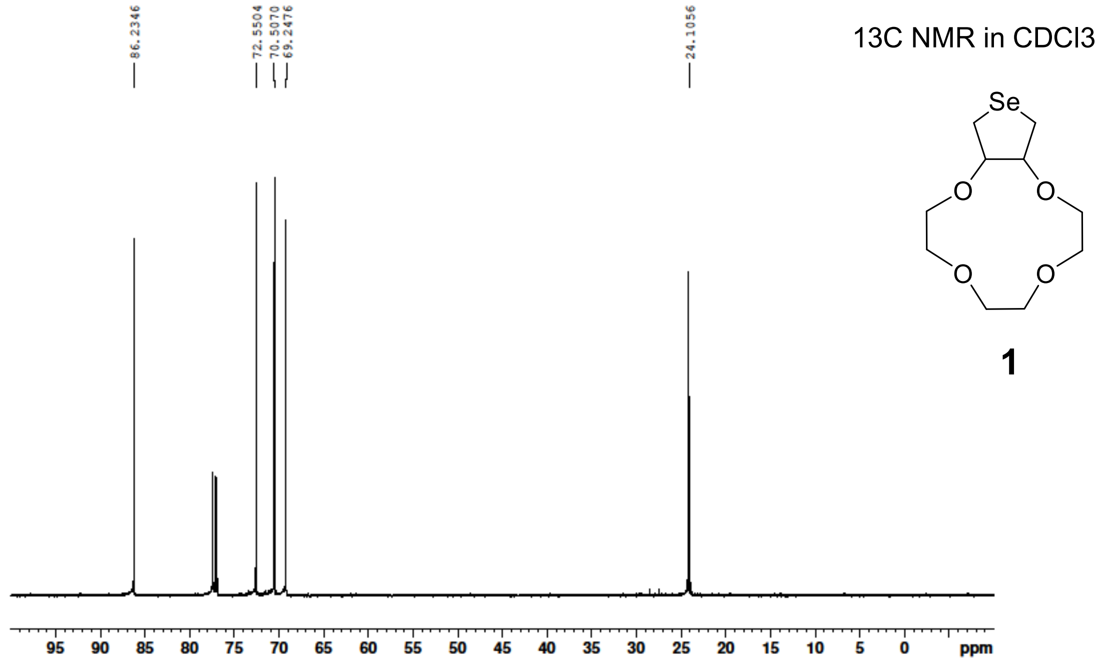

Figure S2. ^{13}C NMR spectrum for DHS-crown-4 (**1**) in $CDCl_3$.

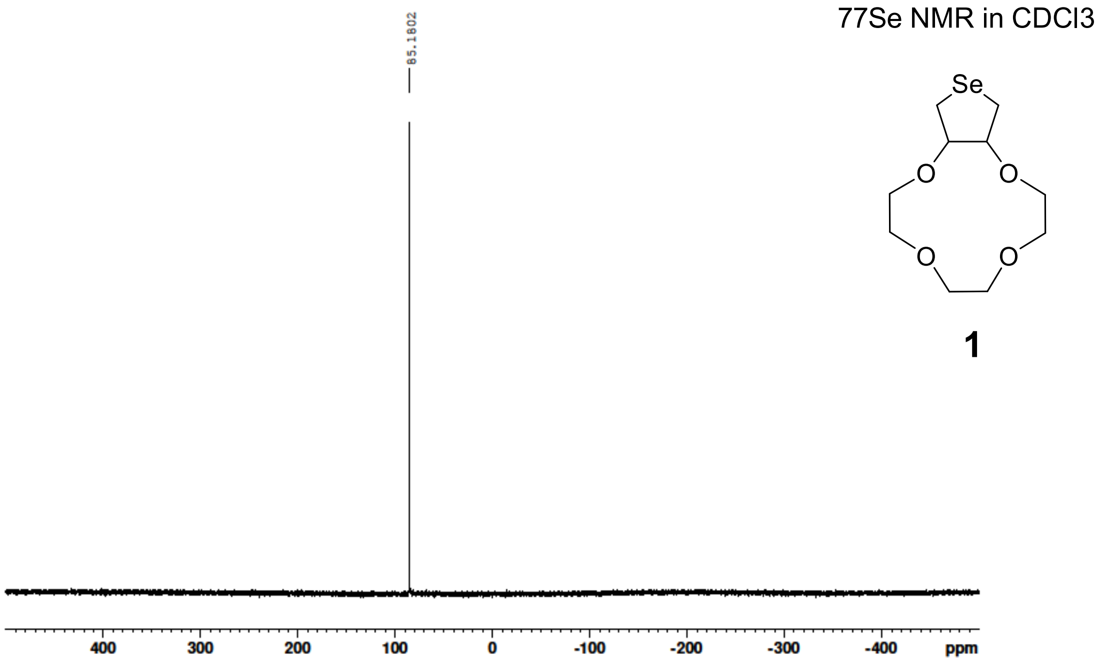

Figure S3. ^{77}Se NMR spectrum for DHS-crown-4 (**1**) in $CDCl_3$.

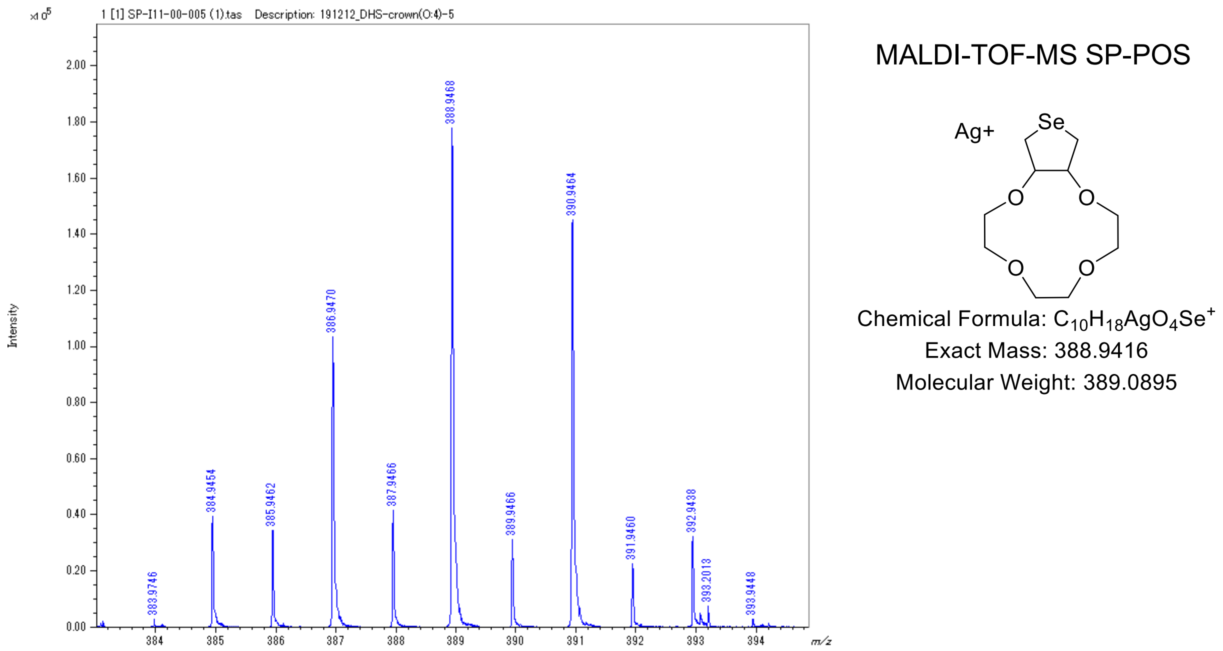

Figure S4. MALDI-TOF-MS spectrum for DHS-crown-4 (**1**)

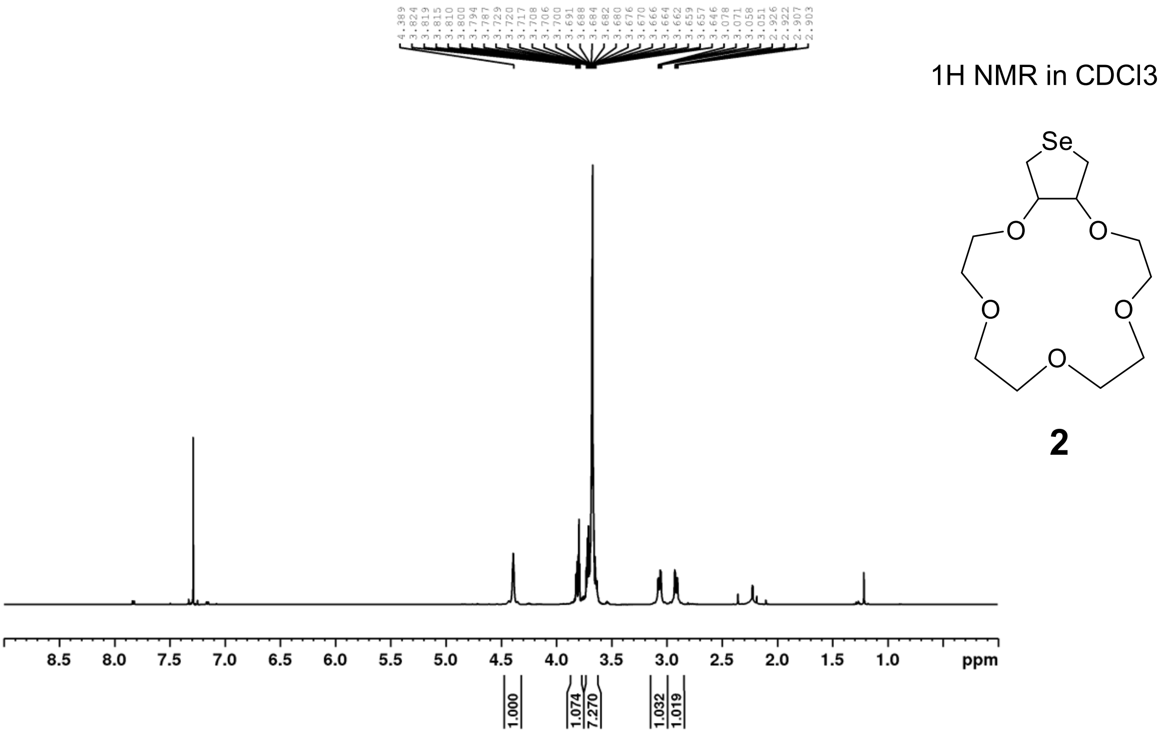

Figure S5. ^{1}H NMR spectrum for DHS-crown-5 (**2**) in $CDCl_3$.

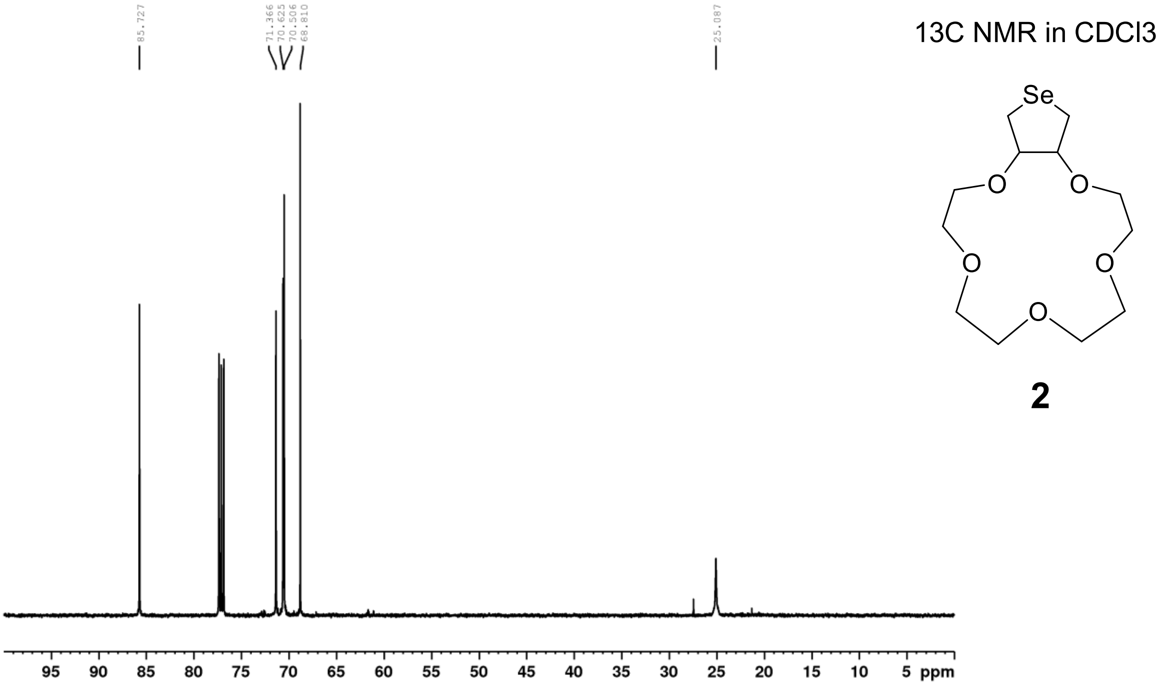

Figure S6. ^{13}C NMR spectrum for DHS-crown-5 (**2**) in $CDCl_3$.

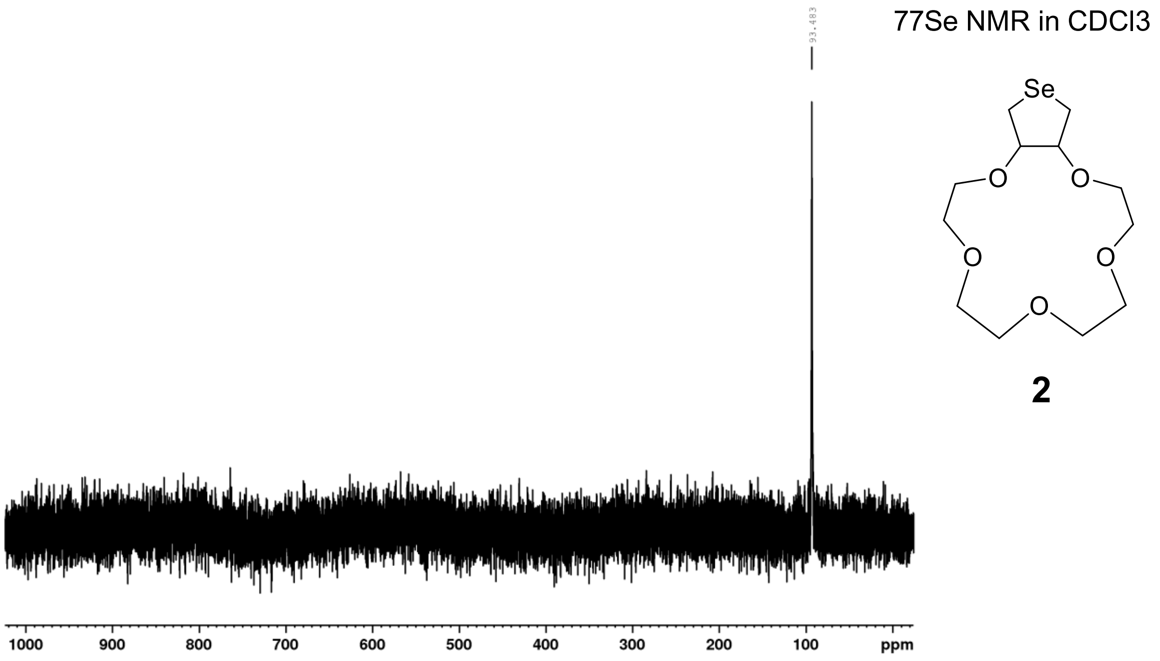

Figure S7. ^{77}Se NMR spectrum for DHS-crown-5 (**2**) in $CDCl_3$.

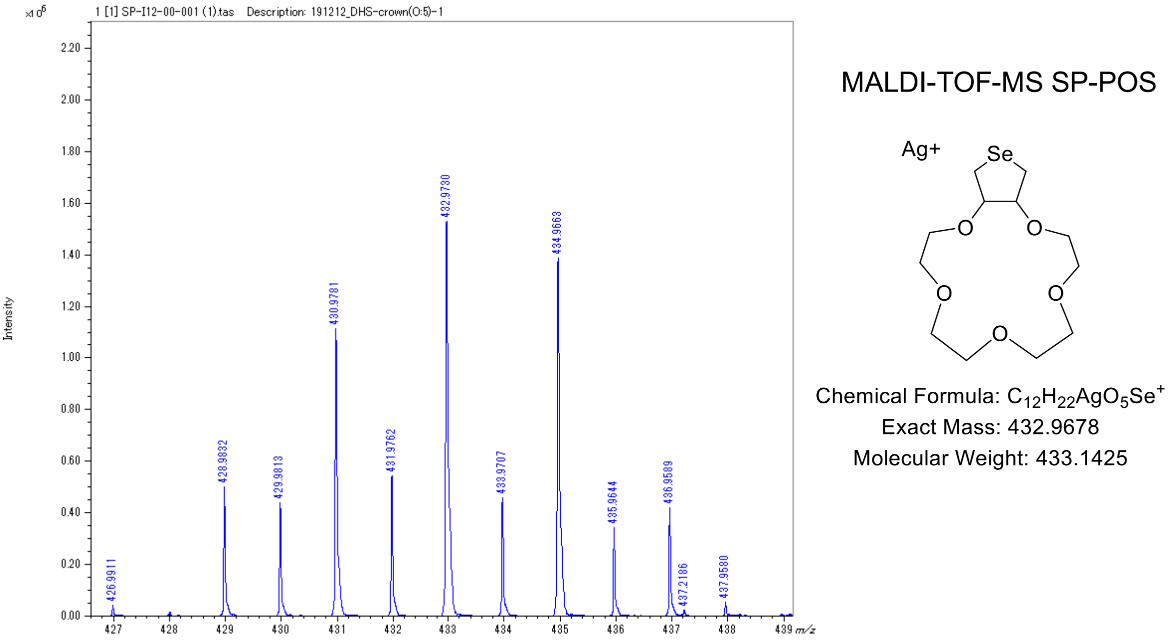

Figure S8. MALDI-TOF-MS spectrum for DHS-crown-5 (**2**)

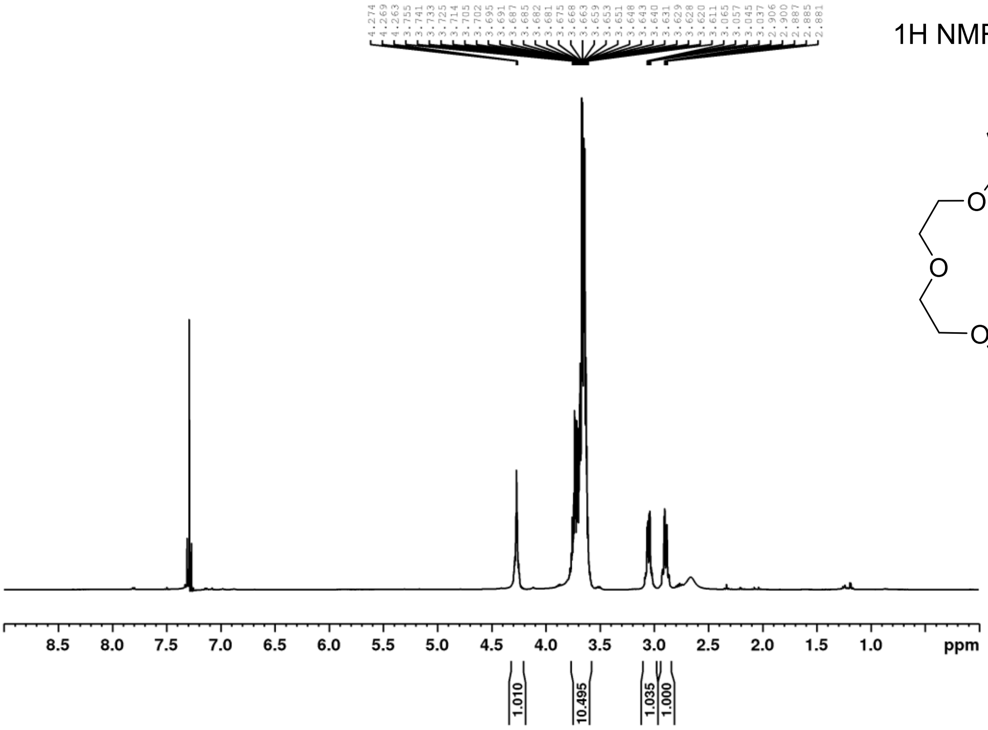

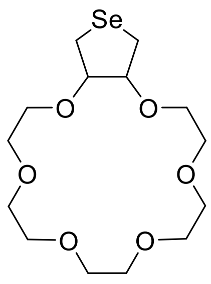

3

Figure S9. ^{1}H NMR spectrum for DHS-crown-6 (**3**) in $CDCl_3$.

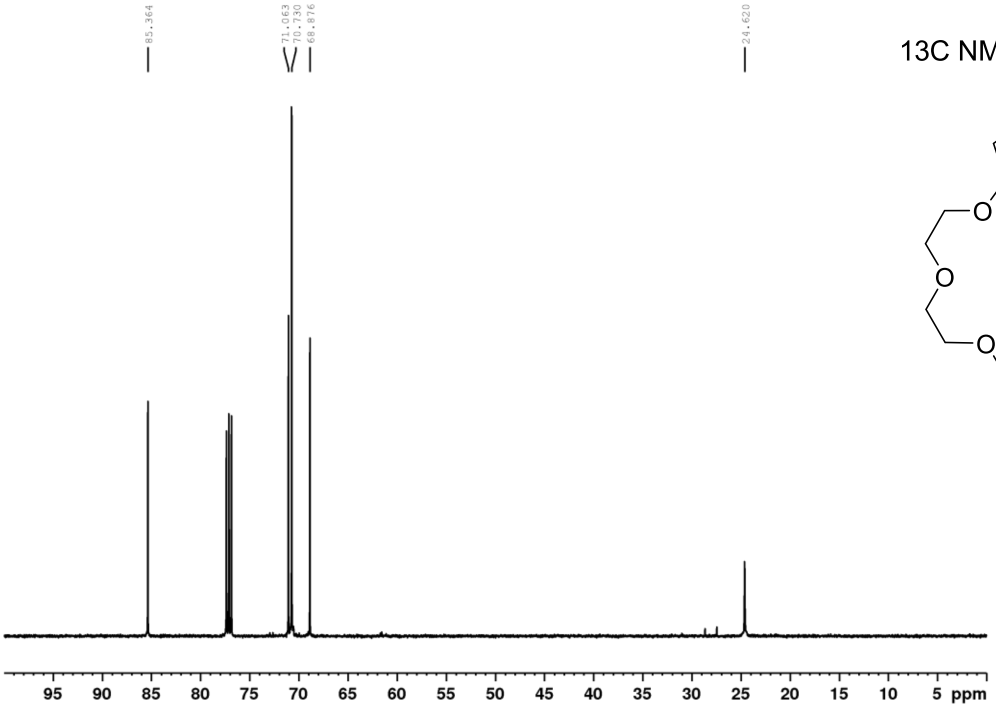

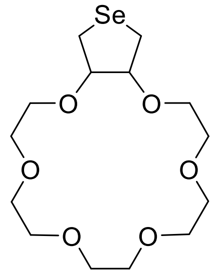

3

Figure S10. ^{13}C NMR spectrum for DHS-crown-6 (**3**) in $CDCl_3$.

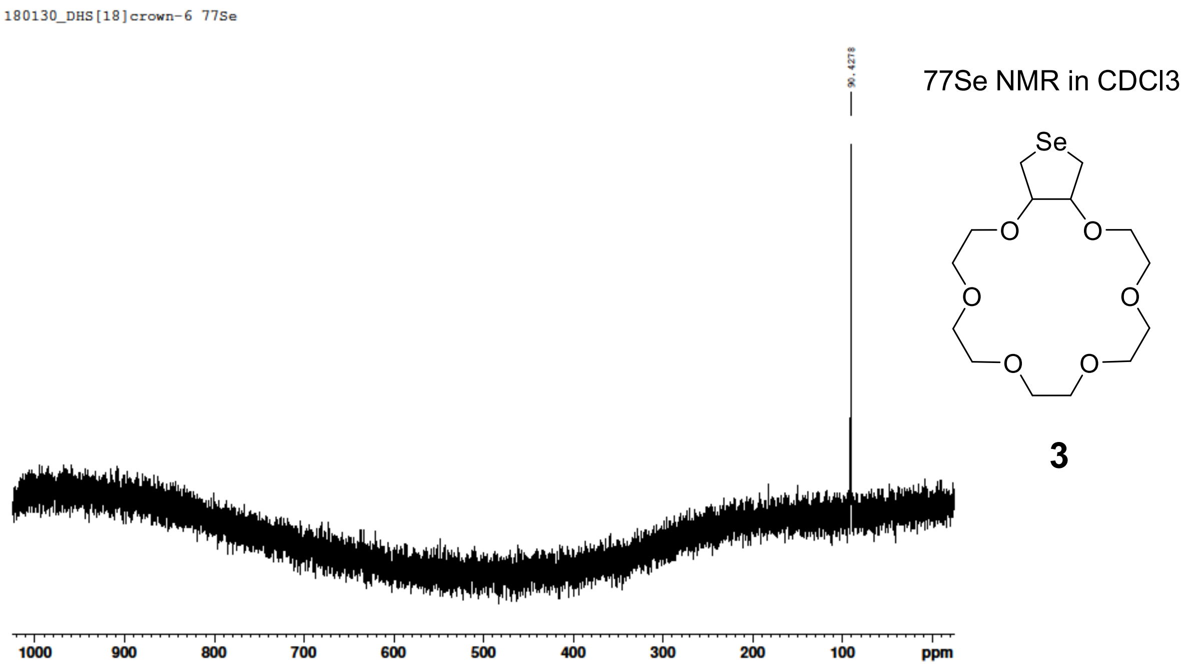

Figure S11. ^{77}Se NMR spectrum for DHS-crown-6 (**3**) in $CDCl_3$.

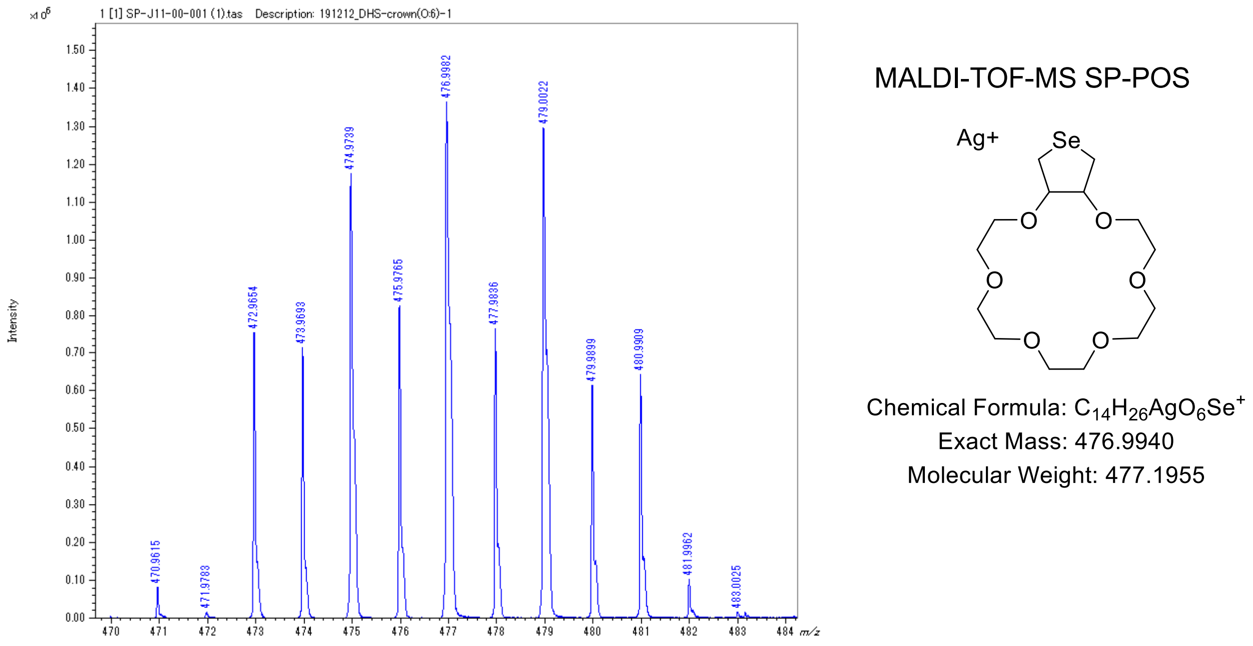

Figure S12. MALDI-TOF-MS spectrum for DHS-crown-6 (**3**).

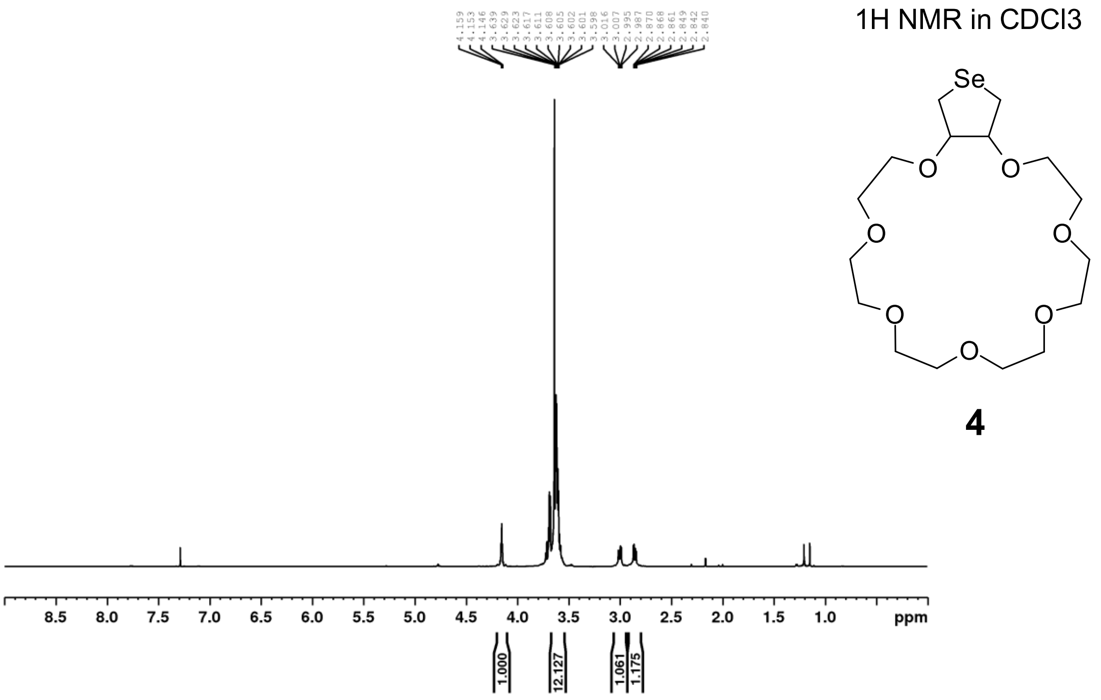

Figure S13. ^{1}H NMR spectrum for DHS-crown-7 (**4**) in $CDCl_3$.

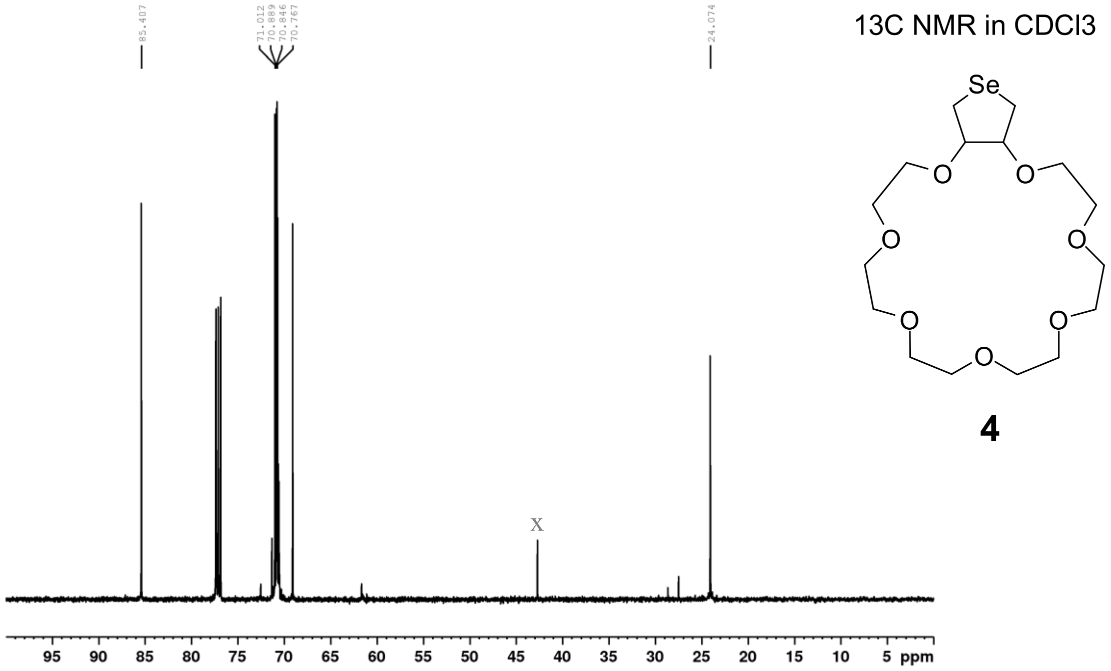

Figure S14. ^{13}C NMR spectrum for DHS-crown-7 (**4**) in $CDCl_3$.

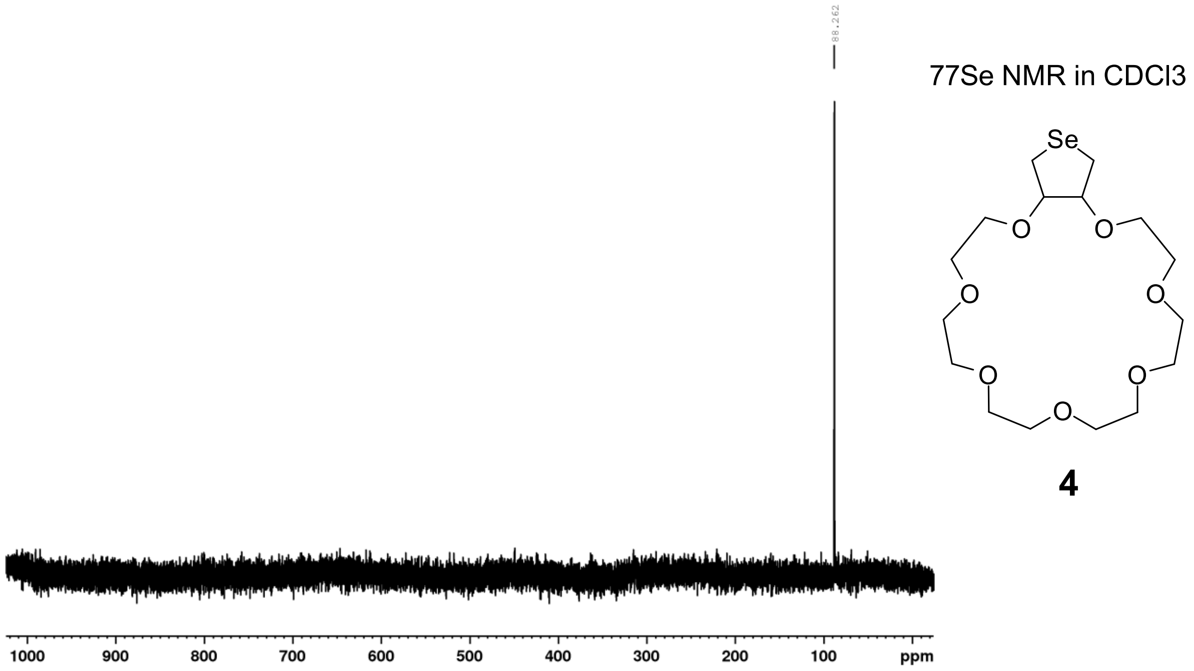

Figure S15. ^{77}Se NMR spectrum for DHS-crown-7 (**4**) in $CDCl_3$.

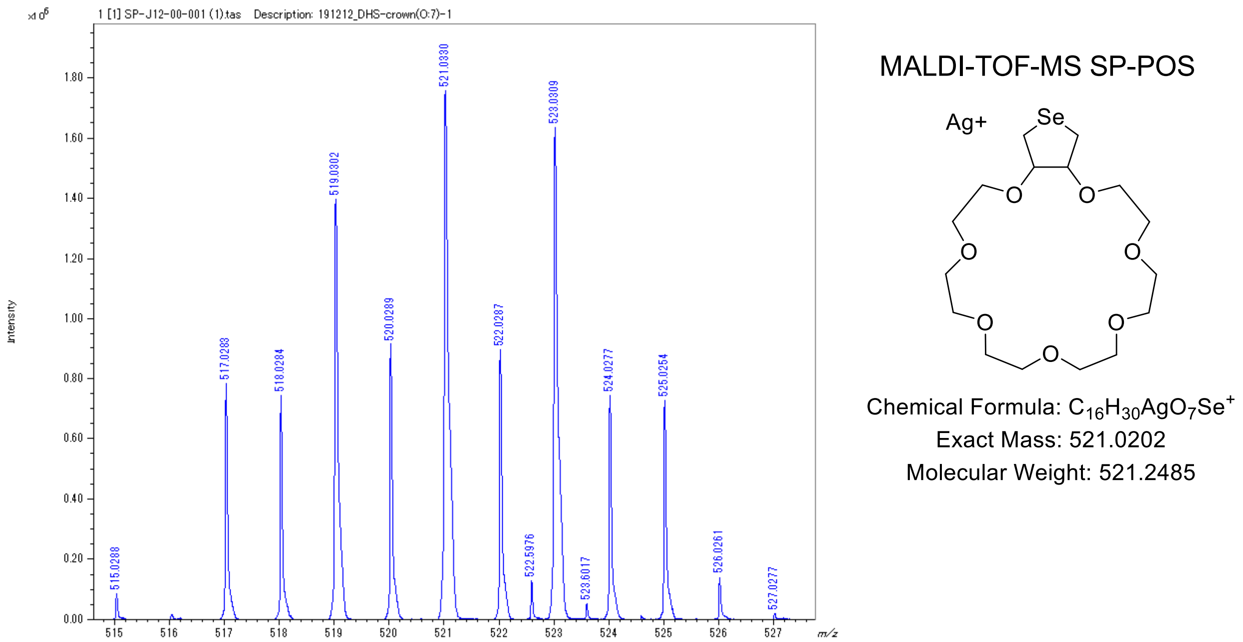

Figure S16. MALDI-TOF-MS spectrum for DHS-crown-7 (**4**).

2. X-ray crystal data for DHS-crown-4 (1), DHS-crown-4·0.5KI·H_2O (5), DHS-crown-4·0.5NaBr·2H_2O (6), DHS-crown-5·0.5KI (7) and DHS-crown-6·KI (8).

Table S1. Crystallographic data for **1**, **5** and **6**.

Compound	DHS-crown-4 (**1**)	DHS-crown-4·0.5KI·H_2O (**5**)	DHS-crown-4·0.5NaBr·2H_2O (**6**)
CCDC Deposition Number	2248791	2248792	2248793
Chemical formula	$C_{10}H_{18}O_4Se$	$C_{20}H_{36}IKO_{10}Se_2$	$C_{20}H_{36}BrNaO_{12}Se_2$
Formula weight	281.21	760.42	729.31
Crystal system	monoclinic	monoclinic	triclinic
Space group	$I2/a$ (No.15)	$C2/c$ (No.15)	$P\bar{1}$ (No.2)
a (Å)	10.7800(12)	13.7060(3)	8.3122(2)
b (Å)	10.5200(12)	13.8760(3)	12.2345(3)
c (Å)	10.7943(14)	14.9320(3)	15.8196(4)
α (°)	90.0000	90.0000	75.5659(19)
β (°)	103.056(6)	100.3900(12)	85.3333(19)
γ (°)	90.0000	90.0000	70.649(2)
V (Å^3)	1192.5(2)	2793.27(10)	1469.98(7)
Z	4	4	2
ρ_{calcd} (g cm^{-3})	1.566	1.808	1.648
T (K)	100	100	103
Wavelength (Å)	0.70000	0.70000	1.54184
μ (mm^{-1})	3.002	3.784	5.362
No. of reflections measured	2112	13002	16821
No. of independent reflections	618	2778	5118
R_{int}	0.0510	0.0360	0.0462
No. of parameters	69	155	325
R_1 ($I > 2\sigma(I)$)	0.0605	0.0637	0.0897
wR_2 (all data)	0.2137	0.1785	0.2326

Table S2. Crystallographic data for **7** and **8**.

Compound	DHS-crown-5· 0.5KI (**7**)	DHS-crown-6· KI (**8**)
CCDC Deposition Number	2248794	2248795
Chemical formula	$C_{24}H_{44}IKO_{10}Se_2$	$C_{14}H_{26}IKO_6Se$
Formula weight	816.53	535.32
Crystal system	triclinic	orthorhombic
Space group	$P\bar{1}$ (No.2)	$P2_12_12_1$ (No.19)
a (Å)	9.4499(2)	10.929(2)
b (Å)	13.4634(3)	13.228(3)
c (Å)	14.0735(3)	13.840(4)
α (°)	82.323(2)	90.0000
β (°)	70.588(2)	90.0000
γ (°)	72.664(2)	90.0000
V (Å^3)	1610.85(7)	2000.8(8)
Z	2	4
ρ_{calcd} (g cm^{-3})	1.683	1.777
T (K)	100	100
Wavelength (Å)	0.71073	0.71075
μ (mm^{-1})	3.431	3.652
No. of reflections measured	24510	14039
No. of independent reflections	8254	4179
R_{int}	0.0472	0.0908
No. of parameters	343	209
R_1 ($I > 2\sigma(I)$)	0.0526	0.0470
wR_2 (all data)	0.1053	0.1107

3. Changes of NMR spectra for DHS-crown-6 (3) in CH_3OD by complexation with KI.

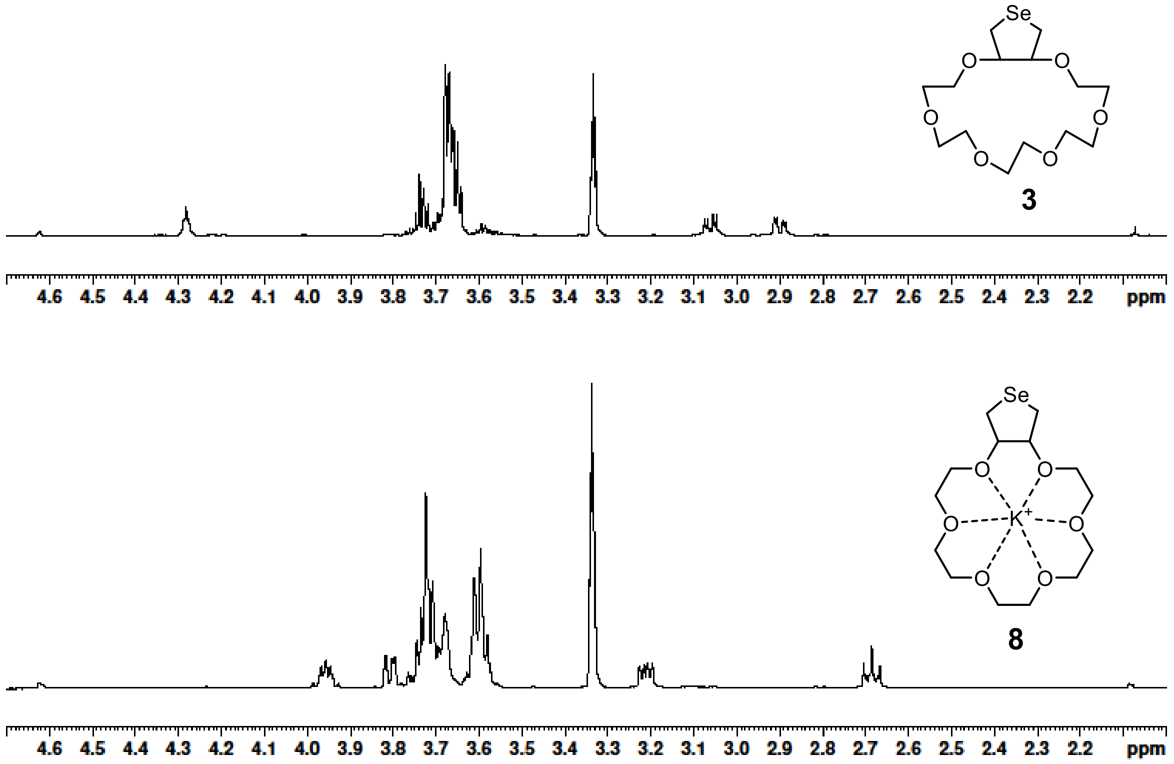

Figure S17. Changes of ^{1}H NMR spectrum for DHS-crown-6 (**3**) in CD_3OD by addition of KI.

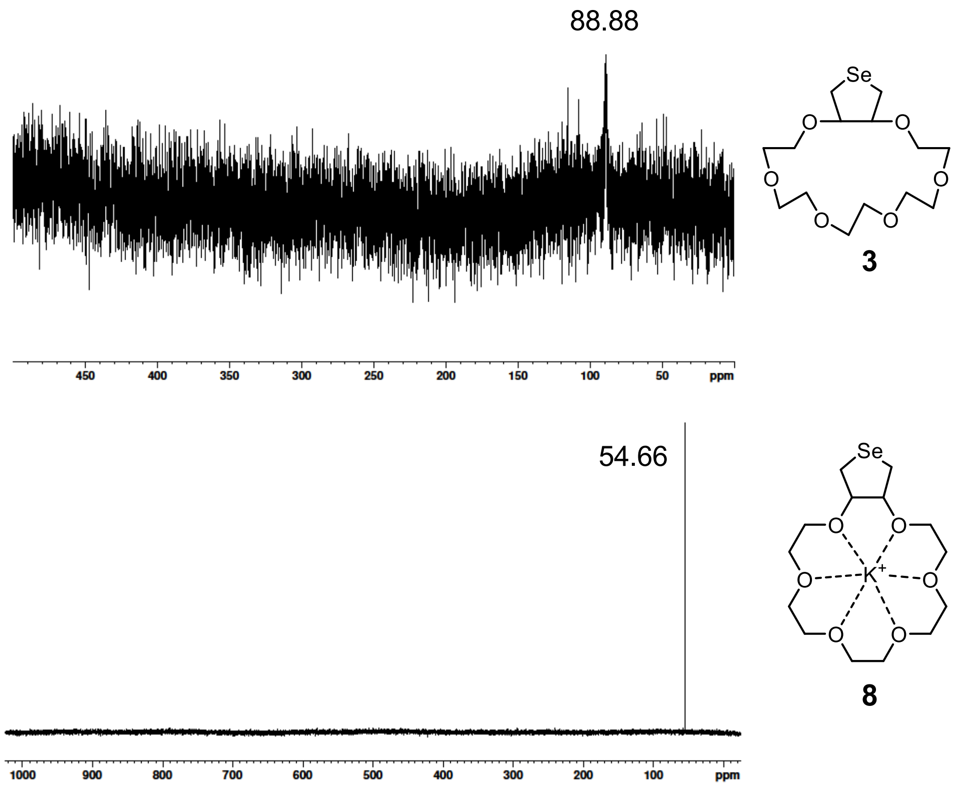

Figure S18. Changes of ^{77}Se NMR spectrum for DHS-crown-6 (**3**) in CD_3OD by addition of KI.

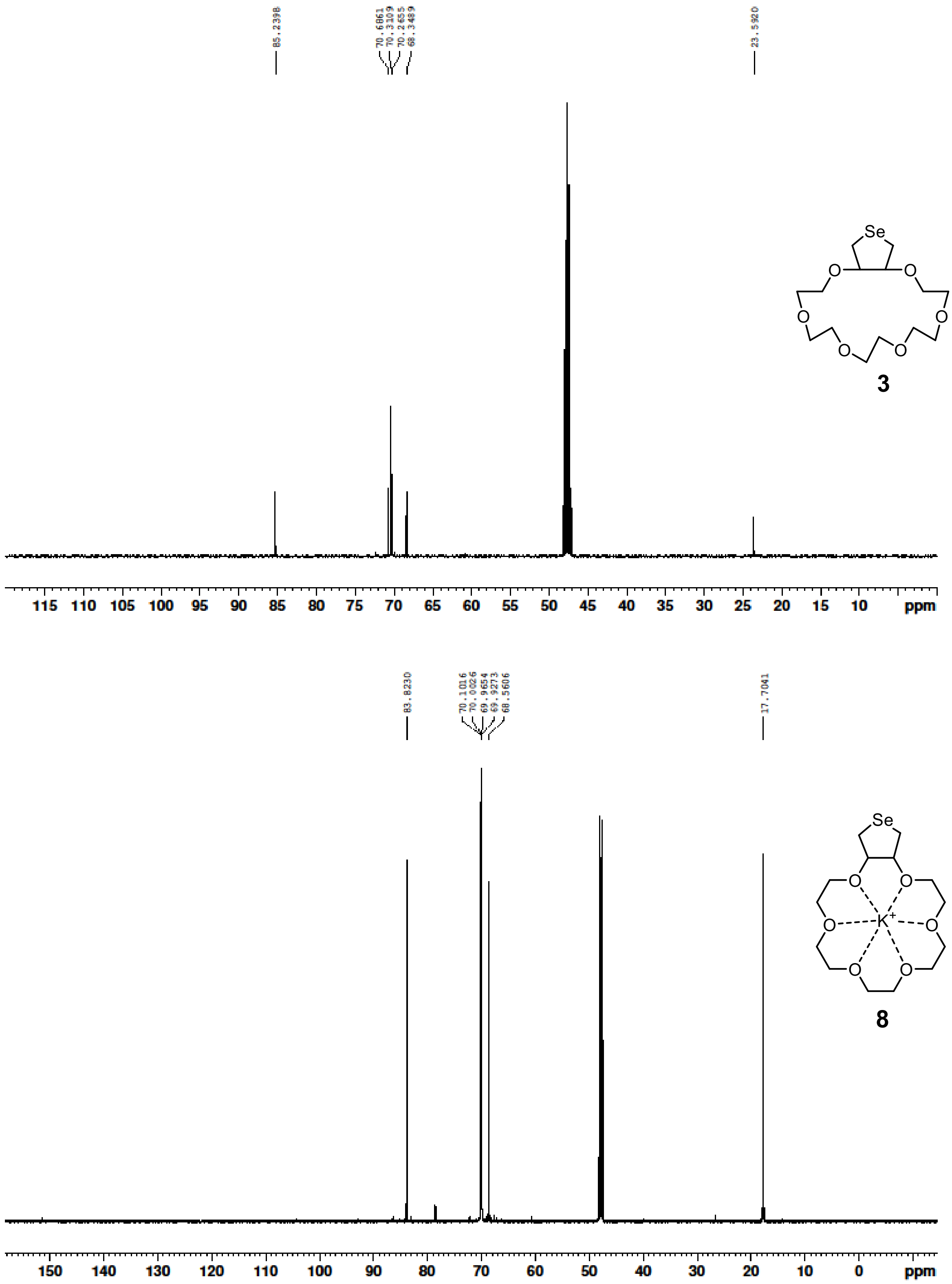

Figure S19. Changes of ^{13}C NMR spectrum for DHS-crown-6 (**3**) in CD_3OD by addition of KI.

4. Series of ^{1}H NMR spectra during ^{1}H NMR titration experiments.

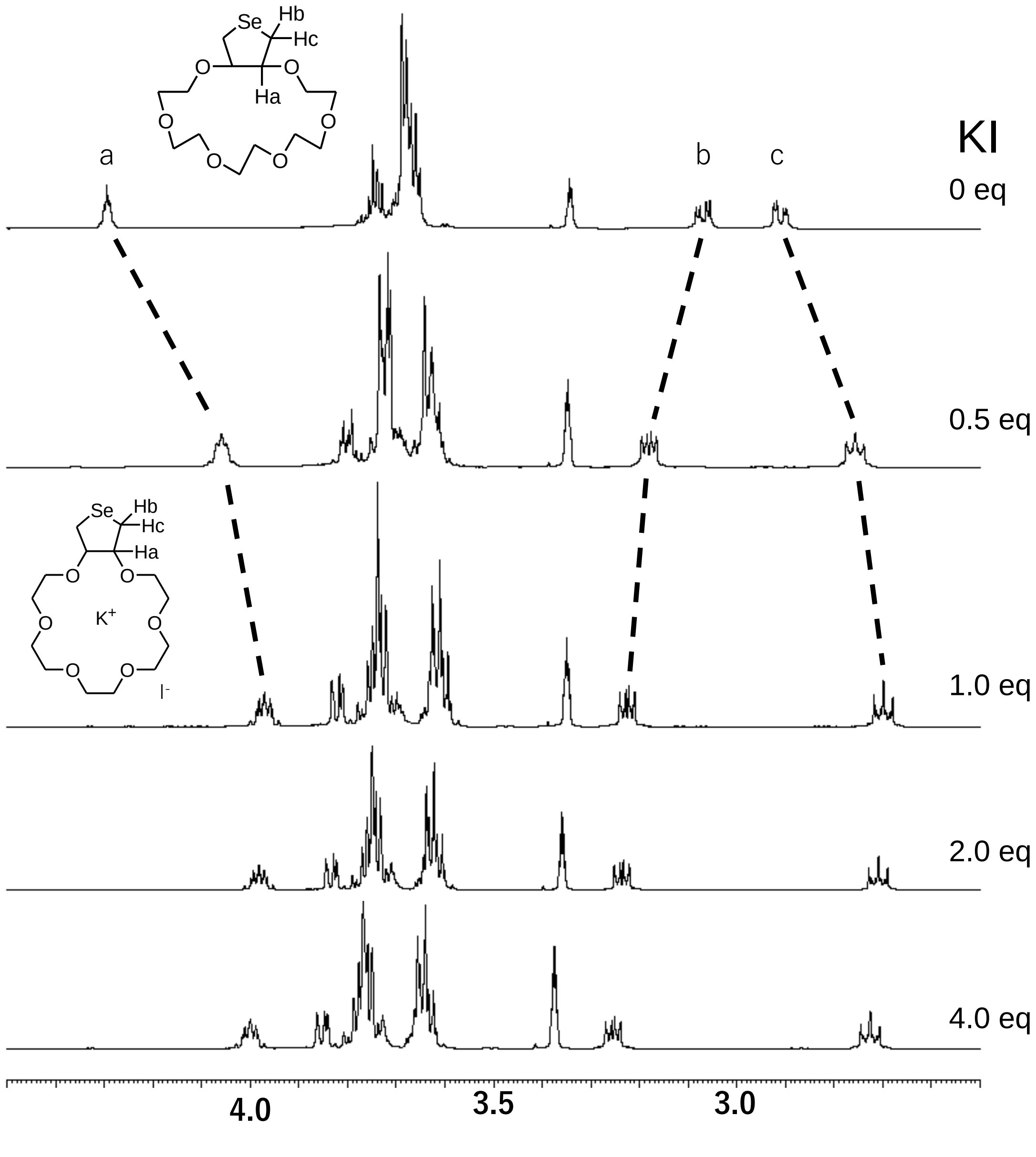

Figure S20. Changes of ^{1}H NMR spectrum for DHS-crown-6 (**3**) in CD_3OD with addition of KI (from 0 to 4.0 eq).

5. Series of ^{1}H NMR spectra during the redox catalytic activity assay for DHS-crown-6 (3) and the KCl complex.

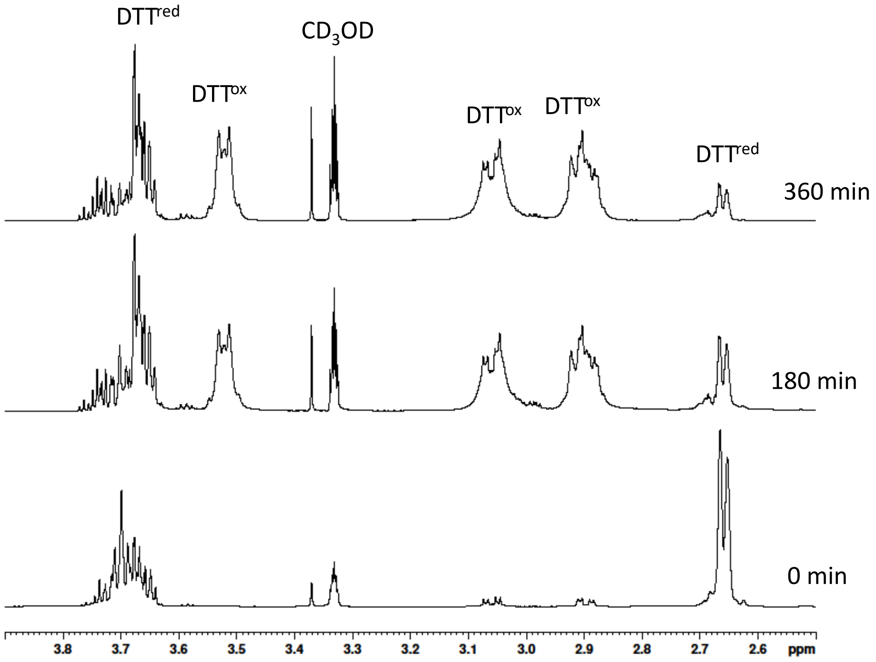

Figure S21. ^{1}H NMR spectra for the mixture of H_2O_2 (0.15 mmol) and DTT^{red} (0.15 mmol) in the presence of DHS-crown-6 (**3**) (0.015 mmol) in CD_3OD (1.1 mL) at 25°C as a function of the reaction time.

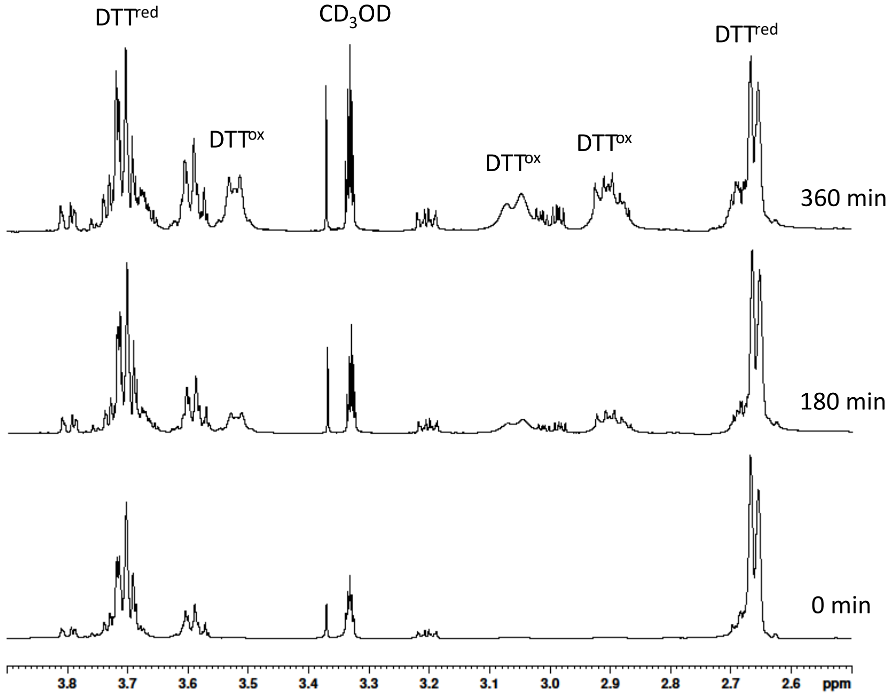

Figure S22. ^{1}H NMR spectra for the mixture of H_2O_2 (0.15 mmol) and DTT^{red} (0.15 mmol) in the presence of DHS-crown-6 (**3**) (0.015 mmol) and KCl (0.015 mmol) in CD_3OD (1.1 mL) at 25°C as a function of the reaction time.

6. ^{1}H and ^{77}Se NMR spectra for the selenoxide of DHS-crown-6 (3^{ox}) and the KCl complex.

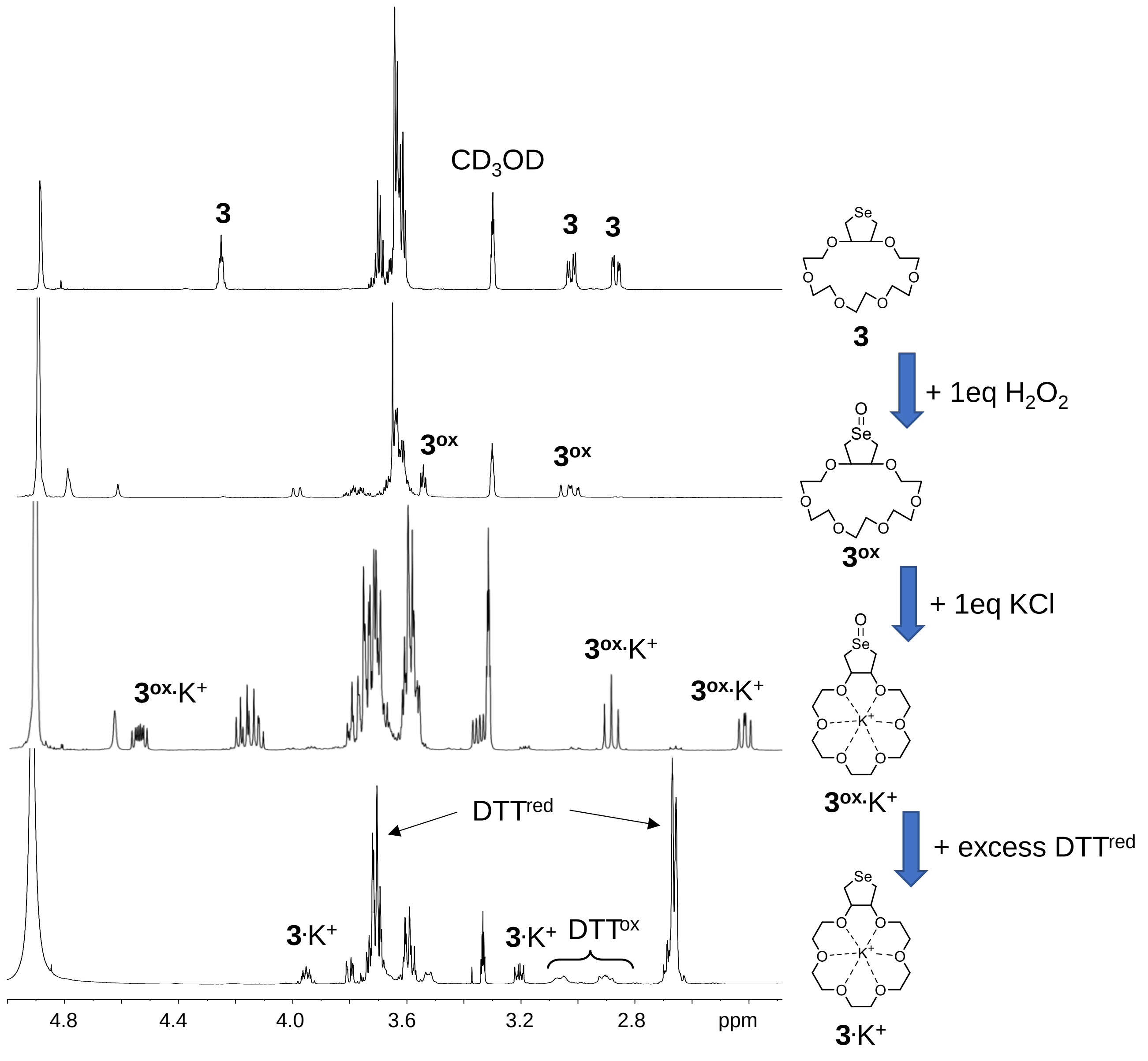

Figure S23 A series of ^{1}H NMR spectra during the formation of the $\mathbf{3^{ox}}\cdot K^{+}$ complex and the reaction with DTT^{red} in CD_3OD.

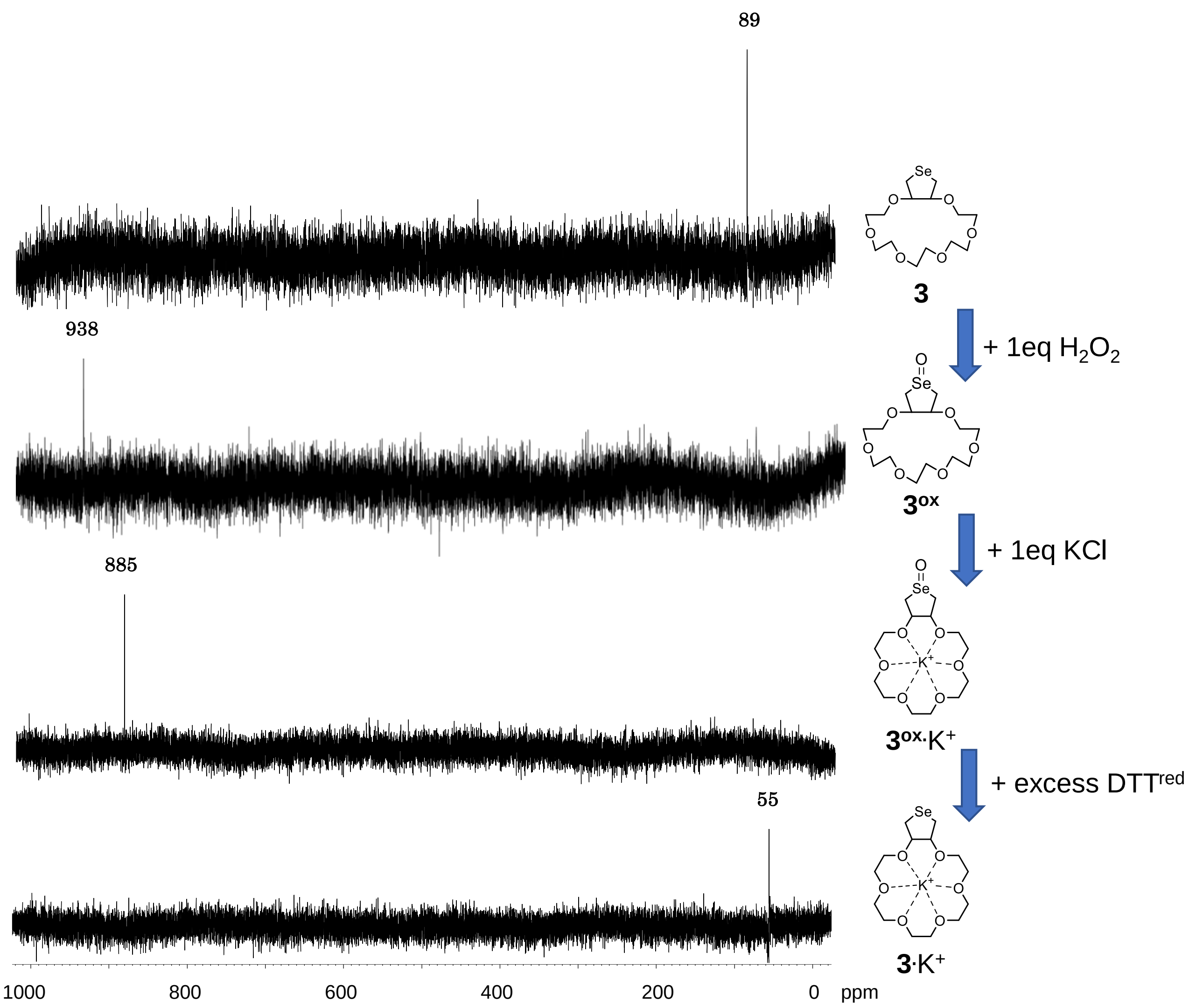

Figure S24 A series of ^{77}Se NMR spectra during the formation of the $\mathbf{3^{ox}}\cdot K^+$ complex and the reaction with DTT^{red} in CD_3OD.

7. Results of DFT calculation.

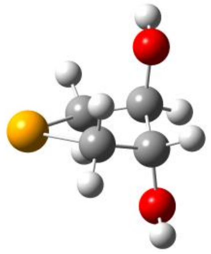

(a)

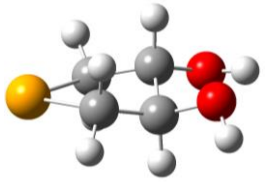

(b)

Figure S25. Molecular structures of DHS obtained by DFT calculation at B3LYP/6-311+g(2df,p) with PCM (solvent = methanol). (a) Diaxial conformer. (b) Diequatorial conformer.

Table S3. Cartesian coordinates for the diaxial conformer of DHS optimized at B3LYP/6-311+g(2df,p) with PCM (solvent = methanol).

Center Number	Atomic Number	Atomic Type	Coordinates (Angstroms) X	Y	Z
1	6	0	0.006188	0.004786	0.024774
2	6	0	-0.005187	0.032820	1.551433
3	6	0	2.471630	-0.015800	0.272656
4	6	0	1.293497	-0.682177	-0.434132
5	1	0	-0.317970	-0.927632	1.954082
6	1	0	-0.654463	0.815840	1.939071
7	1	0	2.739128	0.917694	-0.216691
8	1	0	3.343591	-0.666288	0.311295
9	34	0	1.864895	0.384462	2.121217
10	8	0	1.152890	-2.057928	-0.093013
11	1	0	1.938615	-2.534746	-0.382375
12	8	0	0.024679	1.307858	-0.550104
13	1	0	-0.781759	1.772347	-0.300393
14	1	0	-0.857641	-0.560950	-0.342176
15	1	0	1.398949	-0.567798	-1.518894

Table S4. Cartesian coordinates for the diequatorial conformer of DHS optimized at B3LYP/6-311+g(2df,p) with PCM (solvent = methanol).

Center Number	Atomic Number	Atomic Type	Coordinates (Angstroms) X	Y	Z
1	6	0	-0.005632	-0.009633	0.003073
2	6	0	-0.010800	0.005064	1.526153
3	6	0	2.457956	-0.004919	0.183109
4	6	0	1.275128	-0.704160	-0.461149
5	1	0	-0.000193	1.015820	-0.383628
6	1	0	-0.283840	-0.971058	1.922179
7	1	0	-0.665699	0.767466	1.941279
8	1	0	2.691224	0.920490	-0.339948
9	1	0	3.343642	-0.632788	0.230840
10	1	0	1.231012	-1.748038	-0.121870
11	8	0	1.415162	-0.665319	-1.875827
12	1	0	0.590213	-0.998243	-2.254166
13	8	0	-1.103028	-0.730787	-0.555000
14	1	0	-1.890073	-0.175009	-0.547644
15	34	0	1.866213	0.419873	2.034034
